# A Review of Sisal Fiber-Reinforced Geopolymers: Preparation, Microstructure, and Mechanical Properties

**DOI:** 10.3390/molecules29102401

**Published:** 2024-05-20

**Authors:** Wenbo Qu, Bowen Niu, Chun Lv, Jie Liu

**Affiliations:** 1 College of Architecture and Civil Engineering, Qiqihar University, Qiqihar 161006, China; qwenbo2023@126.com (W.Q.); nbowen2023@163.com (B.N.); 2 College of Light-Industry and Textile Engineering, Qiqihar University, Qiqihar 161006, China; 3 Engineering Research Center for Hemp and Product in Cold Region of Ministry of Education, Qiqihar 161006, China

**Keywords:** plant fiber, GP, sisal, mechanical properties, SFRG, interfacial bonding

## Abstract

The early strength of geopolymers (GPs) and their composites is higher, and the hardening speed is faster than that of ordinary cementitious materials. Due to their wide source of raw materials, low energy consumption in the production process, and lower emissions of pollutants, they are considered to have the most potential to replace ordinary Portland cement. However, similar to other inorganic materials, the GPs themselves have weak flexural and tensile strength and are sensitive to micro-cracks. Improving the toughness of GP materials can be achieved by adding an appropriate amount of fiber materials into the matrix. The use of discrete staple fibers shows great potential in improving the toughness of GPs. Sisal is a natural fiber that is reproducible and easy to obtain. Due to its good mechanical properties, low cost, and low carbon energy usage, sisal fiber (SF) is a GP composite reinforcement with potential development. In this paper, the research progress on the effect of SF on the properties of GP composites in recent decades is reviewed. It mainly includes the chemical composition and physical properties of SFs, the preparation technology of sisal-reinforced geopolymers (SFRGs), the microstructure analysis of the interface of SFs and the GP matrix, and the macroscopic mechanical properties of SFRGs. The properties of SFs make them have good bonding properties with the GP matrix. The addition of SFs can improve the flexural strength and tensile strength of GP composites, and SFRGs have good engineering application prospects.

## 1. Introduction

Geopolymers (GPs) have excellent properties, which will help them become a new type of cementitious material instead of ordinary Portland cement. Compared with traditional cement materials, GPs consume less energy during the production process and emit significantly fewer pollutants. In addition, its hardening speed is faster than that of traditional cement, and its early strength is also higher than that of cement-based materials [1]. In general, the preparation of GPs requires at least two types of raw materials. One is a precursor containing an active aluminosilicate component, and the other is a NaOH or KOH solution containing silicate, which acts as an activator in GPs [2]. The active inorganic Si-Al materials are geopolymerized with the activator to produce a Si-Al gel with a 3D structure. This gel has a spatial network structure composed of silica–aluminum tetrahedral units [3]. A geopolymer is a kind of inorganic polymer material with a three-dimensional network structure composed of Si-O_4_ and Al-O_4_ tetrahedral units. Geopolymers are a kind of environmental cementitious material with low energy consumption and less pollutant emission in the production process. Geopolymers are used in many fields ranging from aeronautics and civil engineering to the plastics industry. Geopolymers are considered green building materials that can replace traditional Portland cement due to their low energy consumption, low carbon emissions, and superior mechanical properties compared with traditional cement [4,5,6,7]. Geopolymers are formed by the polymerization of an active silica–aluminum material with an alkaline activator solution. The synthesis of geopolymers requires an active solid silicoaluminate precursor and alkaline activator solution. The alkaline activator solution has the functions of a binder, activator, and dispersant. Polymeric aluminum silicate materials are formed by exciting geological minerals with an alkali metal silicate solution under strong alkaline conditions. The inorganic silica–alumina cementing material has a three-dimensional network structure. The commonly used active silicate raw materials include fly ash, refined blast furnace slag, and other wastes, such as red mud, rice husk ash, and some mine tailings [8,9,10]. Geopolymers exhibit good thermal and durability properties, but at the same time, they are brittle, show poor resistance to tensile and flexural loadings, and undergo sudden failure and hence, are not suitable for several structural applications [11,12]. To address this issue, research works have been focused on reinforcing geopolymers with synthetic and natural fibers to increase their ductility and resistance to tensile stresses. The incorporation of natural fibers into geopolymers gives a feasible solution to counter its initial brittle behavior [13]. A fiber can be defined as a hair-like material that is either a continuous filament or a discrete elongated piece similar to thread. Fibers can be broadly divided into natural and human-made ones [14,15].

In the past decades, researchers have studied the performance of GPs and the properties of their raw materials. Silica–alumina precursors of GPs can be produced from industrial by-products, including almost all industrial wastes and natural minerals containing aluminum and silicon, such as fly ash, slag, sludge, clay, and kaolin [16,17]. Because of its good strength, excellent fire resistance, and low permeability, GP matrices have been widely used in engineering. However, due to its ceramic properties, GPs have poor flexural and tensile strength and are very sensitive to micro-cracks [18]. The brittleness problem of GPs can be improved by adding reinforcing materials. The incorporation of fibers into the matrix can effectively improve the tensile strength and toughness of the GP, thus controlling the cracking and ductility of the GP composite and limiting the growth of cracks [19].

The fibers in the matrix improve the flexural strength and tensile strength of GP composites. Due to the low alkalinity of the matrix, fiber-reinforced GPs have better durability than ordinary cement-based composites [20,21]. There is a wide variety of fibers used for GP reinforcement, including human-made and natural fibers. There are many varieties of human-made fibers; steel fibers, carbon fibers, glass fibers, and organic fibers are all human-made fibers, and plant fibers, animal fibers, and basalt fibers are natural fibers [22,23,24]. Almost all fibers have a positive effect on the performances of GP composites. Among them are steel fiber and some synthetic fibers, such as polypropylene, polyethylene, and polyvinyl alcohol. However, the production process of these kinds of fibers can easily pollute the environment and consume a lot of natural resources, which is not in line with the goal of sustainable development [25]. Among the natural fibers, plant fibers are the most widely used. Plant fibers, also known as natural cellulose fibers, have many advantages such as its low cost, light weight, strong adhesion, simple manufacturing process, and biodegradability, attracting more and more researchers’ attention. Because of the excellent characteristics of plant fibers, they are the most widely used natural fibers. Plant fibers, also known as vegetable fibers or natural cellulose fibers, have strong adhesion to the matrix. They have the advantages of a low manufacturing cost, light weight, and biodegradability, and have received more and more attention from researchers [26,27].

Plant fiber-reinforced geopolymers are a kind of GP composite that use plant fibers as reinforcement. These plant fibers can come from a variety of natural plants, such as wood, bamboo, hemp, etc. [28,29]. These plant fibers are widely used in GP matrix composites. Due to the addition of plant fiber as a reinforcement, the composite not only has the excellent properties of pure GPs but also has a higher tensile strength, impact toughness, and weather resistance [30]. Because of their green and renewable characteristics, they have been widely used. The use of these composite materials in the construction sector can reduce the dependence on traditional resources and reduce carbon emissions. The composite materials made of plant fiber-reinforced geopolymers not only meet the functional requirements, but also conform to the concept of sustainable development, and will have broad application prospects in the future [31].

Plant fiber-reinforced geopolymers are a new type of composite material, which combines natural plant fibers with GPs. These materials have many advantages. Firstly, plant fibers as a reinforcement can improve the performances of GPs. Due to the natural strength characteristics of plant fibers, they can effectively improve the bending performance and ductility of plant fiber-reinforced geopolymers, making the composites more reliable and durable in the engineering field. Secondly, plant fiber-reinforced geopolymers are also environmentally friendly. Compared with traditional human-made fibers, the use of plant fibers in the production process not only reduces the dependence on fossil resources, but it also reduces carbon dioxide emissions and they are easy to recycle or they decompose naturally after use [32]. In addition, the composite also has good thermal insulation, fire protection, and sound absorption effects. By adjusting the ratio between the plant fiber and GP and the preparation process parameters, the various properties of the polymer are further improved while maintaining the original function, and plant fiber-reinforced geopolymers have a wide application prospect in the field of construction [33]. As a new type of composite, plant fiber-reinforced geopolymers fit in with the concept of sustainable development and will be given more and more of researchers′ attention. In the future, with the advancement of technology and the continuous expansion of the market demand, these composites will bring more practical value to people in various fields.

Sisal fiber (SF) is one of the most common reinforcements used in GPs. In this paper, the preparation technology and properties of sisal fiber-reinforced geopolymers (SFRGs) were analyzed and discussed based on the sci-tech document on SFRGs in recent decades. The physical properties of SFs and the microscopic morphology of the interfacial bonds of SFs in the GP matrix were studied. The effect of SFs on the macroscopic mechanical properties of the GP matrix was discussed based on the analysis of fiber properties and the micromorphology of GPs. It includes compressive, flexural, and tensile strength, and reveals the mechanism of SFs against SFRG matrix cracking.

Plant-derived fibers are themselves well-designed hierarchical composite materials composed of cellulose, hemicellulose, lignin, pectin, waxes, and some water-soluble materials. The structure of a single plant fiber mainly consists of the following constituents: a lumen and a central cavity, which is responsible for the water uptake behavior of the plant, as well as several wall layers, which are grouped into primary and secondary walls. The primary wall is comprised of cellulose microfibrils with a random orientation to allow for the expansion of the cells during the growth of the plant. The secondary wall is subdivided into three sub-layers; cellulose microfibrils in the secondary wall layer present a defined orientation with a helical winding pattern. Briefly, the cell walls consist of cellulose microfibrils coated with hemicellulose structures, which are embedded in a matrix of hemicellulose and lignin cellulose, which is essentially composed of glucose units linked in long chains. These are referred to as elemental fibrils, which are joined to form microfibrils. The cellulose component is composed of crystalline and amorphous regions wherein the crystalline zone is related to the core of the microfibril, while the amorphous zone is associated with the microfibril exterior. The literature reports that cotton, hemp, curaua, jute, pineapple, ramie, and flax fibers contain the highest percentage of cellulose (70–96%), whereas bamboo, bagasse, and coir fibers exhibit the lowest cellulose content (20–45%). Hemicellulose is a complex group of polysaccharides (mainly glucose, mannose, galactose, xylose, and arabinose) and is considered as a mediator between cellulose and lignin. Hemicellulose is covalently linked to lignin and bonded to the microfibril cellulose via hydrogen bonds. Hemicellulose is generally amorphous and contains the highest proportion of accessible OH groups in the cell wall, which is associated with the capacity to increase moisture and a lower thermal stability. It is generally agreed that hemicellulose confers viscoelastic properties to the plants, as its degradation results in an increase in stiffness and brittleness. Lignin is completely amorphous and is composed of a complex group of hydrocarbon polymers with aliphatic and aromatic components. The structure is responsible for the stiffness and the height of the plant and it protects against microbiological attacks and is a chemical adhesive between cell walls in the middle lamella region. Lignin’s mechanical properties are lower than those of cellulose and hemicellulose. Pectin is a collective name for heteropolysaccharides. Pectin confers flexibility to the plants and is predominantly found in the leaves and fruits. Pectin is soluble in water in the presence of alkaline environments with ammonium hydroxide. Finally, fats, waxes, and lipids, which consist of, among other components, different types of alcohols, are insoluble in several solvents as well as in water at room temperature. They serve as a protective barrier against microbiological attacks and prevent the drying process in plants. In general, their presence negatively affects the processing, quality, and wettability of natural fibers [34].

## 2. Properties of SFs

Many factors that affect the compressive strength, flexural strength, and other mechanical properties of geopolymers have been reported by different researchers [35,36,37,38,39,40]. By adding fiber reinforcement to GPs, the plasticity of the composite can be improved to reduce the propagation of matrix micro-cracks and restrain the occurrence of brittle behavior. Plant fibers have attracted the attention of researchers because they are a renewable material and there is a low cost to obtain them. The properties of these fibers are affected by many factors, such as the type of fiber, the characteristics of the fiber, the aspect ratio of the fiber, and the fiber content [41]. Each type of fiber can fulfill some specific functions of composites. Compared with synthetic fibers, plant fibers have the advantages of low cost, an abundant supply, and they are renewable. These characteristics are the advantages of plant fibers as a GP reinforcement [42].

### 2.1. Characteristics of SFs

SF is a representative plant fiber that has been used to strengthen cement-based and GP composites in recent years. The sisal plant is a perennial tropical and subtropical leaf fiber crop. It is one of the most widely cultivated plants in the world. The tensile properties, fracture strain, and Young’s modulus of SFs are not completely consistent along the fiber length [43]. The fiber characteristics of sisal from different sources and different places are shown in Table 1. Sisal can grow rapidly in a very short time. SFs are derived from the leaves of the sisal plant, which is widely distributed and cultivated in almost all regions of the world [44]. Sisal plants usually have 200–250 leaves, each with at least 1000–1200 fiber bundles [45]. Sisal plants consist of fiber, cuticle, dry matter, and water [46]. Among the commonly used fiber plants, SFs account for 2% of the global plant fiber production [47].

It can be seen from Table 1 that SFs, which are commonly used for GP reinforcement, have good tensile strength and a high modulus. The physical properties of SFs are slightly different from those of other plant fibers commonly used for GP reinforcement, as shown in Table 2.

### 2.2. Chemical Composition of SFs

**Figure 1:**
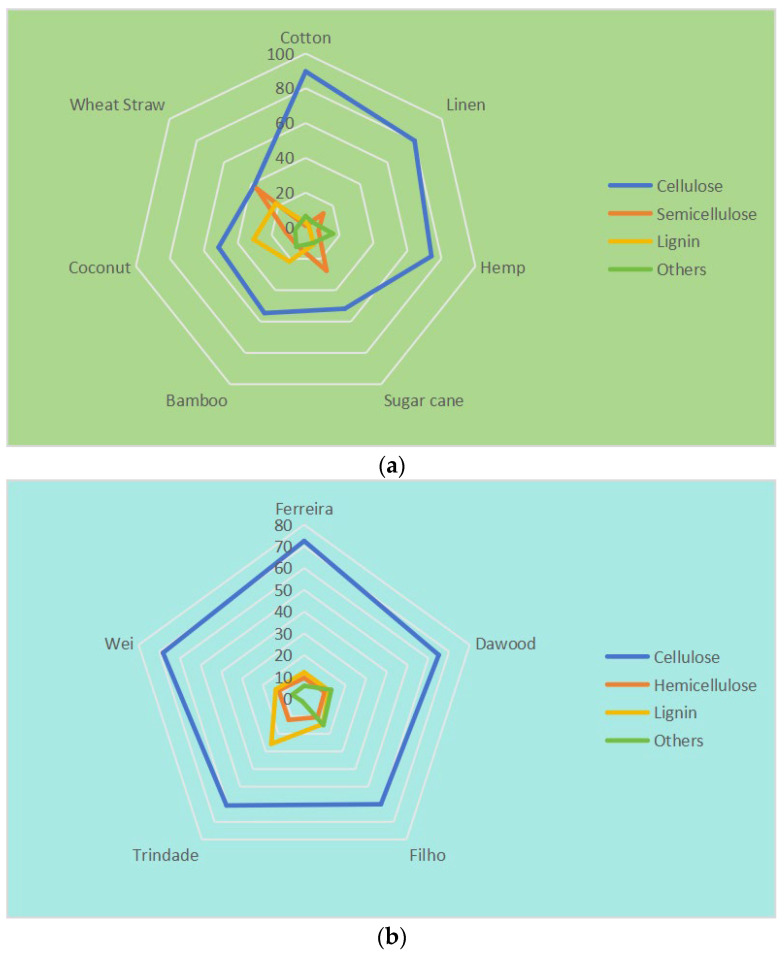
Radar plot for the percentage of plant fibers in chemical composition. (**a**) Different plant fibers; (**b**) SFs.

Plant fiber is mainly composed of 40–60% cellulose, 20–40% hemicellulose, and 10–25% lignin, while the content of other impurities such as sugars, waxes, and other impurities is relatively small [33]. Figure 1a shows the contents of these three main components in different plant fibers [61]. It can be found in the figure that the cellulose content of cotton is the highest, reaching 89.7%, while the cellulose content of wheat straw is the lowest, at only 38.0%, which is almost equal to the hemicellulose content. One of the main limitations of the use of plant fibers in a cement-based material matrix is the alkaline degradation of components such as lignin and hemicelluloses in highly alkaline environments. This not only reduces the bonding ability between plant fibers and the cement matrix but also leads to a decline in the long-term performance of the composites [62,63]. The cellulose content of SF is 60–70%, as shown in Figure 1b [50,64,65,66,67]. It can be seen that the cellulose content of SF is higher than most other plant fibers, but lower than cotton fiber, higher than wheat straw fiber and bamboo fiber, and similar to bast fiber such as hemp.

**Figure 2:**
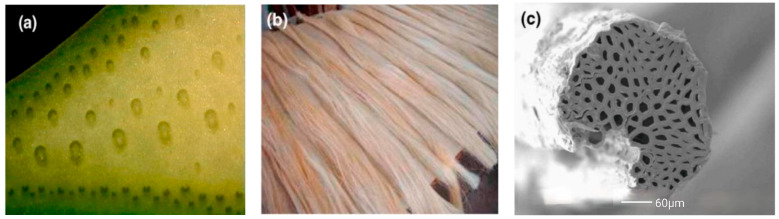
Sisal plants and their fibers. (**a**) Sisal leaf; (**b**) SFs; (**c**) microstructure of SF. Adapted with permission from [49], copyright 2021 Elsevier.

The chemical composition of SFs is also slightly different due to the different origins, varieties, and treatment methods. Like other plant fibers, SFs contain cellulose, hemicellulose, and lignin, but they also contain a small amount of fructose, wax, and ash. Joseph et al. [68] found that the significant differences in the chemical content of SFs were due to different sources, ages, detection techniques, and other factors, which mainly depended on the age and origin of the plant. SFs contain a large number of slender fiber cells. These fiber cells are bonded together by an intermediate lamella composed of hemicellulose and lignin. Figure 2 shows sisal plants, fibers, and their microstructure. The sisal leaves in Figure 2a show the distribution of SFs. Figure 2b shows SFs after extraction and cleaning; Figure 2c shows the microstructure of an SF composed of several fiber cells [49]. It can be seen that inside the SF, there are several micropores of different sizes.

Because of its inherent advantages, plant fibers are more popular than synthetic fibers in the reinforcement of composites [69]. The comparison of cement-based composites with and without plant fibers showed significant differences in the main strength parameters [70]. Adding SFs to the composite matrix can slow down the hydration process of the matrix and prolong the setting time. Correia et al. [71] added 3% volume and 25 mm length SFs to a GP matrix to adjust the performances of the composite.

SFs are produced from the hard leaves of the sisal plant and are leaf fibers. Azevedo et al. and Li et al. summarized SFs by comparing them with synthetic fibers [72,73]. Firstly, SFs are low in cost, rich in sources, and sisal plants are planted in large quantities all over the world. Secondly, sisal itself can biodegrade and regenerate. Thirdly, the SF production process is pollution-free and environmentally friendly. An SF is made of 100–200 primary fibers bonded by pectin in a layered structure [74]. The SF surface layer has a large number of hydroxyl groups that are easy to combine with water and can absorb a lot of water. In addition, the hollow lumen of each primary fiber also absorbs water through capillary action, which is stored in the lumen [75,76]. This moisture can be used for the self-curing of GPs or cement concrete, which has a good influence on improving the performance of the matrix [77].

## 3. Selection and Preparation of GP Matrices

### 3.1. GP Composites

The main materials in the GP matrix include the precursor and activator. The precursors are industrial wastes or natural minerals rich in silicaluminate. The activator is generally an alkaline activator prepared using a strong alkali solution, and an acidic activator such as phosphate is also used.

#### 3.1.1. Precursor

The precursor raw materials are mainly metakaolin or clay minerals, as well as industrial wastes such as fly ash, slag, and other volcanic by-products. Some researchers also use sludge, red mud, and rice hull ash as precursors [78,79,80]. The precursor usually used for the preparation of GPs in experimental research is metakaolin, fly ash, or a mixture of both materials. Generally, specific fly ash is used as the matrix for the production of GPs [48]. High-dose pozzolanic materials can also be used in matrix production, and the cementing materials are Portland cement, fly ash, and metakaolin to obtain a low-alkaline GP matrix [81]. Dawood et al. [65] used a local ceramic powder and slag in their study. The ceramic powder, cement, and slag are initially mixed, and then the fine aggregate is put into the mixture to prepare the dry mixed mortar.

#### 3.1.2. Activator

The activators include alkaline activators and acidic activators. Alkaline activators are widely used. Alkaline activators commonly used in the preparation of GP include NaOH solutions, KOH solutions, and water glass. Tap water is generally used for deployment. 

#### 3.1.3. Aggregate

The aggregate is generally quartz sand or ordinary medium sand. Sometimes the properties of composites are adjusted by adding nanomaterials [82,83,84]. Castoldi et al. [81] took river sand as the fine aggregate for a GP, and its maximum particle size was controlled within 4.75 mm, and its fineness modulus was 3.02. The maximum diameter of the coarse aggregate used in GPs was 12.5 mm. In addition, the stone powder was added to the GP to improve its performance.

#### 3.1.4. Fiber Reinforcement

The main function of adding fibers into the GP matrix is to increase the toughness of the composite. Traditional fibers used as fiber reinforcement include steel fiber, carbon fiber, glass fiber, basalt fiber, polymer fiber, etc. Plant fibers are also being used more and more widely. Plant fibers are mainly bast fibers, such as flax fibers, hemp fibers, and jute fibers [85,86]. SFs are also widely used leaf fibers. Generally, plant fibers need to be pre-treated before use to improve the performance of the fibers. Castoldi et al. [81] soaked SFs in 70 °C water for 60 min and then air-dried them for 2 days. Finally, the fibers were cut by hand into 50 mm segments and soaked in a sodium hydroxide solution at room temperature for 60 min. The pre-treated SFs had better mechanical properties and durability.

### 3.2. Design of Mix Proportion

According to the different requirements of engineering materials and different experimental research purposes, the mix ratio of materials is designed. Through an analysis of the existing experimental studies on SFRGs, we found that most of them focus on the influence of different fiber contents on the properties of the composites [87]. Therefore, in the design of the mix ratio, the control variable method is used, that is, the fixed water–binder ratio, cementitious sand ratio, the ratio of alkaline activator to cementitious material, and water glass modulus, and the fiber content is changed. The parameters of the mix ratio were determined by trial matching. These parameters include the ratio of each component of the precursor aluminosilicate raw material, binder–sand ratio, and water–binder ratio. The ratio of each component of the precursor silicoaluminate raw material is mainly the ratio of fly ash to metakaolin. The W/B is the ratio of the water content of the external water and alkali activator to the mass of the cementitious material. Castoldi et al. [81] used Portland cement, meta-kaolin, and fly ash in their matrix production; their mass ratios were 50%, 30%, and 20%, respectively, and the water–binder ratio was 0.5. The GP reference matrix used by Trindade et al. [50] is an adhesive activation material composed of metakaolin. The binder to aggregate weight ratio of the matrix was 1:1. Zhou et al. [88] designed a GP with equal amounts of fly ash and slag powder added. In this GP, the content of glass sand accounted for 60%. The cement–sand ratio of the specimen was 0.67, while the liquid–solid ratio was set at 0.75. Different kinds of plant fibers with 1% of the specimen mass were added as a GP reinforcement. The particle size of the ceramic powder and slag used by Dawood et al. [65] was controlled at 0.045 mm. The chemical composition of ceramic powder SiO_2_ and alumina accounted for 63.29% and 18.29%, respectively.

The alkali activator solution is prepared by the geopolymerization of solid sodium hydroxide or potassium hydroxide, water glass, and water. This reaction releases a lot of heat, which requires about 24 h to cool down. When adjusting the modulus of the sodium silicate solution, it is necessary to add solid sodium hydroxide to the solution. The mass fraction of sodium oxide is adjusted by adding water to the solution through calculations and test preparations. Most of the activators are prepared with 12 M sodium hydroxide solution. Dawood et al. [65] dissolved sodium hydroxide in distilled water to prepare a 12 M sodium hydroxide solution. After 24 h, the sodium hydroxide and Na_2_SiO_3_ solution were mixed. The mixture needed to sit for several hours. There were also sodium hydroxide solutions in other moduli. The alkali activator in reference [88] was composed of an aqueous solution of Na_2_SiO_3_ with a modulus of 2.3 and a sodium hydroxide solution of 8.4 M, with a ratio of 2.5:1. The excitant of Korniejenko et al. [48] was an 8 M sodium hydroxide solution combined with a sodium silicate solution at a ratio of 1:2.5. An alkaline solution was prepared by pouring an aqueous solution of sodium silicate and water onto solid sodium hydroxide. The solution was mixed until the temperature was stable and the concentration was balanced. Kavipriya et al. [89] evaluated the properties of the composites by adding 0.25%, 0.5%, 0.75%, and 1.0% SFs, respectively. They used bamboo sticks instead of coarse aggregate. Various parameters were considered in the design of the mixture: the concrete grade was M25, the ratio of fly ash to alkaline activator solution was 0.67, the concentration of sodium hydroxide was 10 M, the ratio of sodium hydroxide to the sodium silicate solution was 1:2.5, and the high-efficiency water-reducing agent was 1% of the mass of fly ash. Korniejenko et al. [48] used 1% of the weight of the composite as a filling for cotton, sisal, raffia, and coconut. The SFRG mix ratio designs of relevant studies are shown in Table 3.

The precursor raw materials of SFRGs are mainly fly ash and metakaolin, as shown in Table 3. It is necessary to ensure the fineness of the precursor materials during preparation so that they can fully geopolymerize with the activator. The main activators are sodium hydroxide solutions and sodium silicate solutions. The weight ratio of the SFRG precursor to activator is generally between 0.6 and 0.8 [90,91].

### 3.3. Preparation Process of SFRG Mortars

**Figure 3:**
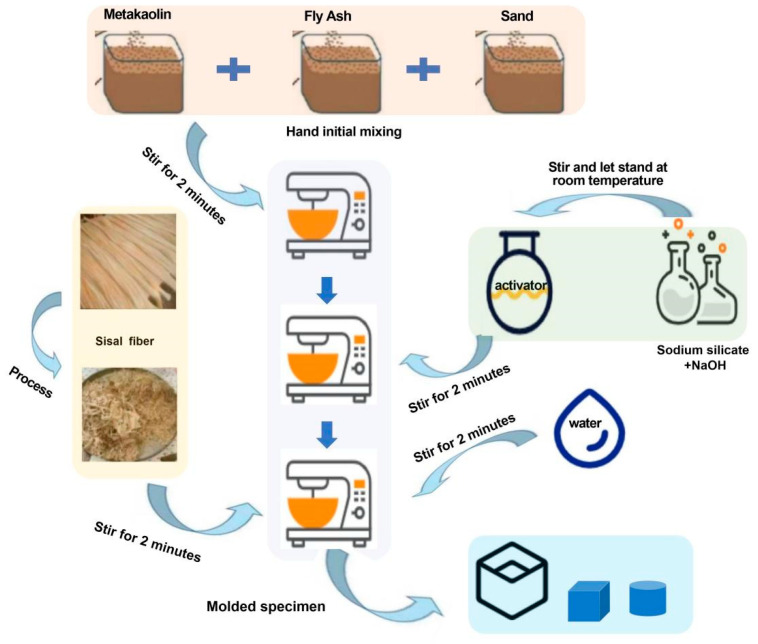
Flow chart of the SFRG preparation process.

When preparing SFRG mortars with different fiber contents, it is necessary to ensure that the fibers can be evenly dispersed in the slurry, so that the strengthening effect and improvement effect of the mortars can achieved. To ensure the achievement of the experimental research objectives, it is necessary to select a suitable preparation method and process. The feeding sequence of GP composite material is different from that of ordinary cement-based composite material. Generally, after the precursor and aggregate are dry mixed evenly, the activator solution, fiber, water-reducing agent, and water are added in sequence, and a forced mixer is used for wet mixing. The stirring duration can be appropriately adjusted according to the performance requirements of the composite material [88,92]. Naghizadeh et al. [93] prepared a base polymer of recovered fly ash. The ratio of aggregate to binder was 2.25. After mixing the dried material with a laboratory mortar mixer for 1 min, the alkali activator solution was added to the mixture for 2 min (activator to adhesive ratio of 0.5). The specific preparation process of an SFRG is shown in Figure 3.

Due to the lack of a separate standard for the preparation process of GP materials, it is generally recommended to refer to the ordinary concrete strength test standard and select multiple specimens [12,94]. The precursor material, aggregate, alkaline activator solution, and SFs are added and stirred successively in the concrete mixer to form a slurry with good workability. It is then poured into the specimen mold. The mold is generally heated and cured in the laboratory for 24 h and then cured in the environmental mode or standard mode for 28 days. Castoldi et al. [81] used a concrete mixer to prepare a GP slurry. After 24 h of casting, the specimen was removed from the mold and placed in a curing box with a temperature of about 21 °C and a relative humidity (RH) of 100% for 28 days. Ferreira et al. [95] used the test standard based on ASTM C191-19 [96] to prepare their product in the laboratory at a temperature of 21 °C and RH of 60. At the end of the mixing process, the filling mold was vibrated on a shaking table. According to DINEN196-1 [97], the cast size of the specimen was 160 × 40 × 40 mm and 10 × 10 × 10 mm. The geopolymer specimen was cured in a mold, sealed with a plastic film, and cured in the laboratory. Mechanical tests were carried out on the 28th day after curing. The main technical indexes of the preparation process of SFRGs are shown in Table 4.

## 4. Properties of SFRGs

### 4.1. Microscopic Morphology

The mechanical properties and thermal stability of the composite can be significantly improved by adjusting the bond between the plant fibers and GP. Through a microscopic level analysis, the microscopic interface between the plant fibers and the matrix can be confirmed to be well bonded, which can effectively improve the macroscopic mechanical properties of the composite [33].

#### 4.1.1. Scanning Electron Microscopy (SEM)

**Figure 4:**
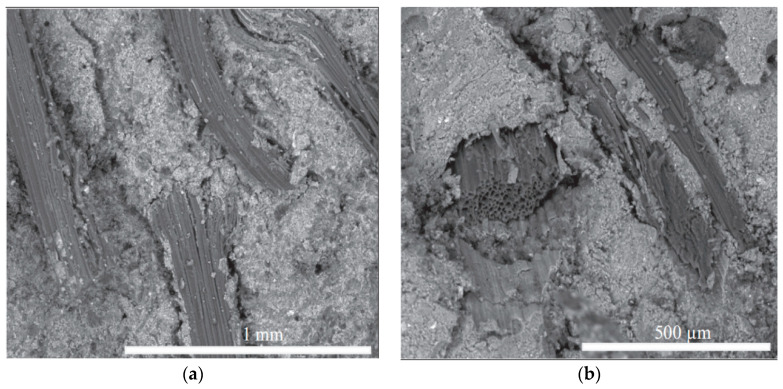
SEM micrograph of fractured FRGC. (**a**) Fiber–matrix binding; (**b**) failure mechanism. Adapted with permission from [101], copyright 2023 Elsevier.

The microstructure of SFRG composites is complex, and the bonding between the interfaces affects the macroscopic performances of SFRG composites. Therefore, it is also very important to study the microstructure of GP composites. The influence of different types of fibers on the interfacial bonding properties with the GP matrix is very different. Eco-friendly materials like plant fibers and geopolymer binders play a key role in the path toward sustainable construction. In general, fibers pulling out rather than fiber breaking is more conducive to improving the ductility of the matrix. Therefore, good bonding between the fibers and the matrix is a necessary condition to avoid fiber breakage, thereby improving micro-cracking behavior and ductility [98]. The microstructure of composites can be studied by SEM. The analysis of the micromorphology of the composite material provides preliminary information on the coherence of the natural fibers with the GP matrix and evaluates the distribution of the fibers [99,100]. The distribution of the fibers in the GP matrix seriously affects the properties of the SFRG, because the aggregation of fibers will reduce its mechanical properties [101]. Korniejenko et al. [48] observed the sections of a compression test by SEM at different magnifications and found that the distribution of plant fibers in the SFRG was regular. In the same specimen, it is found that some fibers were agglomerated and unevenly distributed, which led to the degradation of the composite’s properties. Similarly, the scanning electron microscope images of Silva et al. [13] showed that there was excellent adhesion between the SFs and GP, as shown in Figure 4a. On the other hand, the decrease in compressive and tensile strength observed with 3% SF content may be caused by poor compaction and fiber aggregation due to more fibers, as shown in Figure 4b.

In general, fiber fracture and withdrawal from the matrix are the two main failure mechanisms of composite materials. Wei et al. [102] extracted slices from specimens after a bending test and analyzed the microstructure of the fracture surface of the SF wiener specimen. It can be seen that after 28 days of hydration of the cement matrix, both the cement strong alkali matrix and the SFs produced dry shrinkage, and a void was found around the fiber, indicating a defect caused by mineralization or corrosion. However, the drying shrinkage of fibers in the low alkali solution of the kaolin matrix was reduced, and the fibers still maintained a good initial form. This is also the reason why the flexural strength of the SF-reinforced cement-based specimens decreased and the bending row of the kaolin specimens did not change much after accelerated aging.

**Figure 5:**
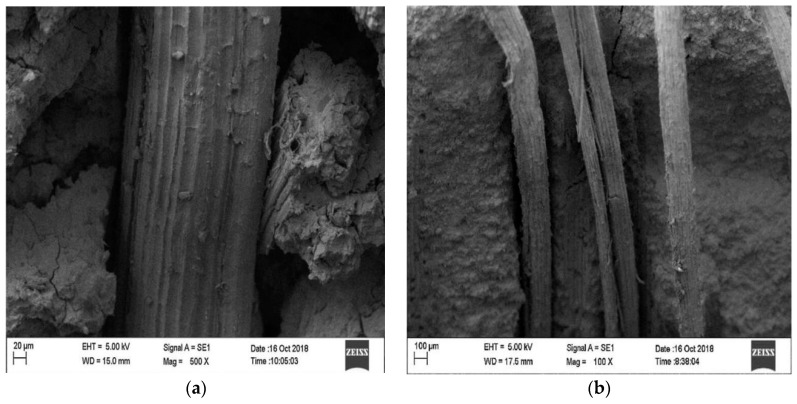
Interface bonding between SF and matrix. (**a**) Adhesion between fibers and matrix. (**b**) The fiber remains intact after the specimen is broken. Adapted with permission from [48], copyright 2016 Elsevier.

Coir fibers and SFs have a similar adhesion to the matrix in terms of micromorphology. Zhou et al. [85] observed coir-based composites by SEM and found that coir fibers had a good compatibility with the GP matrix. The surface of the composite material was smoother than that of the GP without the addition of fibers. Lin et al. [103] observed through a microscopic analysis that the fibers in the bent specimen did not separate and break. He et al. [104], considering the adhesion of the matrix to the fibers, applied a force to the specimen to extract them during the analysis and sampling process using SEM, which resulted in damage to the GP matrix and the brittle rupture of the matrix and fiber interaction. Based on this, Silva et al. [48] used SEM to analyze the properties of the SFs and matrix after bending and breaking, and the specimen was gold-plated. A microscopic analysis by dispersive energy spectrum and SEM can more accurately verify the composition of the matrix [105]. The researchers observed a rough surface for the fibers, indicating adhesion between the fibers and the GP matrix, as shown in Figure 5a. SFs have a serrated surface with sharp peaks and guttering, which makes the interface between the fiber and the matrix adhere better. Ferreira et al. [106] also found, through SEM, that the plant fibers had a zigzag surface in the transverse direction, with repeated crest and trough sequences. The surface of the SFs had an obvious peak groove, while the surface of the jute fibers was flat and smooth. It can be observed from Figure 5b that the fibers remained intact even after the specimen was broken, indicating that the fiber strength was greater than the bond strength between the GP matrix and the fibers. Similar to Silva et al. [48], Duxson et al. [107] and Gao et al. [108] also observed the presence of unreacted silica particles in the entire GP matrix through dispersive energy spectrum analysis of dark particles. This may affect the results of the mechanical strength of the composite material, as the strength of the unreacted material is often lower than that of the ground polymers. The researchers used ratios of activator to metakaolin of 0.69 and 0.748 and found that the unreacted particles still existed after 24 days of curing. With the same fiber content and a ratio of activator to metakaolin equal to 0.55, it was found that the material-dissolved silica particles needed more than 14.5 days of curing time, and almost completely dissolved in 28 days. This suggests that the presence of the unreacted material may be due to the presence of excess silicon ions in the mixture.

Observing from the perspective of the microstructure, the mechanism of plant fiber reinforcement can be obtained. When the matrix without fibers is subjected to an external force, micro-cracks are more likely to occur due to the irregular shape and particle size of fly ash and slag. With the continuous increase in the external load, the matrix micro-cracks continue to extend and expand along the groove, and the specimen is more likely to crack from inside the matrix to outside the matrix, resulting in brittle damage to the GP. After the hydration and gel reaction of the matrix, there will be many honeycomb surfaces inside the matrix, which further aggravates the damage. In addition, the reaction time of GPs is short and the alkali activation reaction is more intense. The formation of a stable Si-Al tetrahedron in a short time will hinder the whole process of polymerization. The microscopic analysis showed that there is a chemical interaction between the plant fibers and inorganic polymer interface, and the failure kinetics of the geopolymer composite included crack bridging, fibers pulling out, and a fiber tearing mechanism. When fibers are added to the composite, the alkali ions in the NaOH solution will ionize a large number of hydroxyl groups in the plant fibers, which makes the plant fibers coarser and dense within the GP slurry. In the gelation process of SFRGs, the adhesion between the plant fibers and matrix is higher. At this time, the gel can fill the holes in the matrix, and the microscopic surface of the SFRG is smoother. Due to the relatively closed overall structure of the matrix, sufficient water is kept inside, so that the alkali concentration of the matrix is always in a relatively stable state during the polymerization of the matrix, and the mechanical properties are not reduced. Sisal fibers have strong water absorption characteristics. The water-saturated SFs are evenly mixed with the GP matrix, ensuring that the matrix is always in a humid environment. In this way, the reaction time of SFRG hydration and gel is prolonged, and the geopolymerization of SFRGs will be more thorough [88].

#### 4.1.2. Thermogravimetry

**Figure 6:**
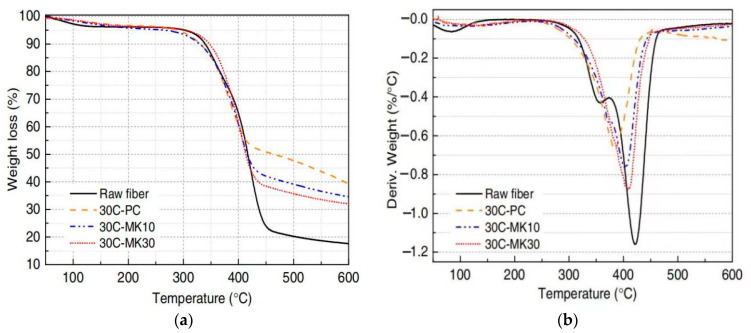
Thermogravimetric analysis curves of raw SFs and fibers in different environments. (**a**) Thermogravimetric analysis; (**b**) derivative thermogravimetry.

The key feature of thermogravimetry is that it is highly quantitative and can accurately measure the mass change rate of SFs in GPs using thermogravimetric analysis and derivative thermogravimetric analysis. The information on the weight loss ratio, temperature, and decomposition residue of SFs can be obtained through analyses to understand the interface bonding property between SFs and the GP matrix. Wei and Meyer [100] used a thermogravimetric analyzer to determine the components immersed in SFs under different environments at heating rates ranging from room temperature to 700 °C. Sisal raw fibers and SFs in a cement matrix (30C-PC), a 10% metakaolin matrix (30C-MK10), and a 30% metakaolin matrix (30C-MK30) after 30 dry–wet cycles were analyzed. The results of thermogravimetry of the SFs under different environments are shown in Figure 6a. Sisal raw fibers and the SFs in MK30 began to undergo an obvious degradation process at 300 °C. The degradation process of the fibers pulled from the MK10 and PC matrices was not obvious at 280 °C and 260 °C. After 30 dry–wet cycles between room temperature and 600 °C, the thermal degradation behaviors of raw SFs and fibers embedded in the PC, MK10, and MK30 matrices were similar. Due to the evaporation of water in the fibers, the weight of the fibers decreased slightly from 50 to 110 °C; at 270–350 °C, the weight of the main part decreased due to the decomposition of hemicellulose and lignin. The maximum weight loss occurred at high temperatures with the degradation of cellulose and the remaining lignin. Studies have shown that the wide degradation range of lignin components of SFs in two or three stages makes thermogravimetric analyses more difficult [109,110]. In addition, the derivative thermogravimetry curve of the specimen shows the displacement ranges of the fibers with different degradation degrees at each stage, as shown in Figure 6b. The main decomposition step of sisal raw fibers occurs at 260 °C to 490 °C, while the main decomposition step of SFs in the MK30 matrix occurs at 270 °C to 460 °C. At this stage, the cleavage of the cellulosic glycosidic bond reduces its degree of geopolymerization, resulting in the formation of carbon dioxide, water, and various hydrocarbon derivatives [111,112]. The thermal stability of SFs is slightly different in different environments.

Wei et al. [113] also studied the overall thermal decomposition process of SFs in another study. It was found that a distinct peak was observed in each specimen due to the decomposition of cellulose. At 350 °C, it was usually caused by the thermal decomposition of hemicellulose, while the decomposition of lignin did not result in any significant peak.

#### 4.1.3. X-ray Diffraction (XRD)

**Figure 7:**
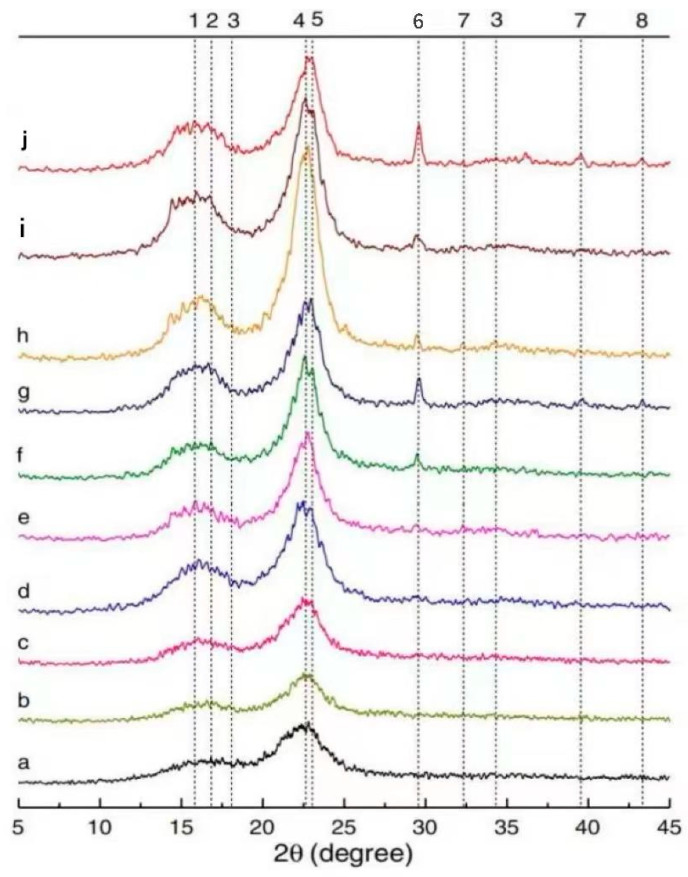
XRD pattern of SFs in different environments. Adapted with permission from [86], copyright 2021 Elsevier.

The qualitative and quantitative analyses of mineral phases in composites can be achieved using XRD. The grain size of SFs in the GP matrix can be calculated using the XRD spectrum to characterize the crystallinity level of SFs after long-term aging [12]. Wei et al. [102] analyzed the XRD patterns of SF-reinforced 10% metakaolin and 30% metakaolin specimens after different dry/wet cycles. In Figure 7, a~j represent SFs embedded in cement matrix after 5 dry/wet cycles, SFs embedded in 10% metakaolin matrix after 5 dry/wet cycles, SFs embedded in 30% metakaolin matrix after 5 dry/wet cycles, SFs embedded in cement matrix after 15 dry/wet cycles, SFs embedded in 10% metakaolin matrix after 15 dry/wet cycles, SFs embedded in 30% metakaolin matrix after 15 dry/wet cycles, SFs embedded in cement matrix after 30 dry/wet cycles, SFs embedded in 10% metakaolin matrix after 30 dry/wet cycles, and SFs embedded in 30% metakaolin matrix after 30 dry/wet cycles, respectively. As can be seen in the figure, cellulose has crystalline properties, and there is a dense peak corresponding to the lattice plane at 2θ ≈ 22.5°. The ettringite peaks were present in all SFs except the raw fibers, while calcium hydroxide peaks were detected only in SFs immersed in the cement matrix. Due to the pozzolanic activity of metakaolin and its dilution in the matrix, calcium hydroxide and ettringite are consumed, and the calcium ion concentration is greatly reduced. As a result, the hydrated products of calcium hydroxide and ettringite precipitate less in the lumen of SFs. The 29.2° peak was dominated by C-S-H and calcite, which can be detected in 15 wet/dry cycles and became more prominent after 30 wet/dry cycles. It is believed that this is caused by the residual cement hydrate on the surface of SFs. The degradation of SFs in the matrix is intensified with the increase in the number in dry/wet cycles.

Trindade et al. [50] found that the XRD results were related to aluminosilicate materials, which showed a clear quartz peak, while the content of muscovite, kaolinite, and illite was low. A semi-crystalline behavior is dominant in the GP diffraction pattern. Trindade and Silva et al. [29] noted that all the matrices showed similar crystal behaviors through the XRD patterns of the GP matrix. It was detected that the crystal peak of impurities found in metakaolin was dominant. It can also be seen that the amorphous structure of silica powder did not significantly change the crystallization behavior of the GP. Huang et al. [114] found through XRD spectra that the XRD spectra of the corresponding specimens were similar in different environments after the dry/wet cycles, and the detected crystals were all calcite. This indicates that the specimens under different external environments are significantly affected by carbonation [115,116]. Ranjbar et al. [56] reported that an XRD pattern analysis showed that the main diffraction peaks of fiber-reinforced GPs were the same as those of a common matrix, indicating that the influence of fibers on the geopolymerization process was not significant.

### 4.2. Mechanical Properties of SFRGs

The microstructure of fiber-reinforced GPs affects its macroscopic properties. Many studies have shown that the addition of plant fibers to the matrix has a significant effect on the mechanical properties of GPs. The results of compressive, flexural, and splitting tensile tests showed that the mechanical properties of GP composites can be improved by the proper addition of plant fibers.

#### 4.2.1. Compressive Performance

The compressive properties of cement-based composites are one of the important components for measuring their mechanical properties. The compressive performance of SFRGs mainly depends on the compressive capacity of the matrix and is also closely related to the interface bonding between the matrix and fibers [117,118]. The influence factors of plant fibers on the compression of GPs mainly include the fiber type and fiber content [59,119]. The effect of SFs on the compressive strength of the GP matrix is not obviously different from that of most plant fibers.

The compactness of the SFRG matrix can be improved by distributing the appropriate amount of fibers uniformly in the matrix, thus reducing cracks and pores, and improving the compressive strength of the composite [33,120]. Varuthaiya et al. [53] summarized the strength properties of conventional concrete, GPs, and SFRGs with different fiber contents. The addition of 0.6% SFs produces a maximum compressive strength of 33.05 MPa within 28 days, which was 12% higher than conventional concrete. Ref. [81] also found that the compressive strength of SFRG specimens after 28 days was 35.0 MPa. According to the test results, the compressive strength decreased steadily after the proportion of SFs increased to 0.6%. Similarly, Fujiyama et al. [121] also found that mixing SFs into slurry had little effect on the compressive strength of the GP. When the fiber content was 1.0%, the performance of the GP was improved. Zhou et al. [88] tested the compressive performance of different plant fiber-reinforced geopolymers. The growth trend of the compressive performance of the plant fiber-reinforced geopolymers was consistent with that of pure GP. The compressive performance of GP composites containing plant fibers was better than that of pure GP. At the three different ages of 7, 14, and 28 days, the compressive strength difference remained at about 20 MPa. The specimens containing coir fibers had the best performance, followed by those containing jute fibers and SFs. The mechanical properties of the other plant fiber specimens were the same.

The results of the compression test by Trindade et al. [50] showed three different stages in the development of GP strength. The first stage is the first 24 h of curing. Two hours after casting, the specimen is still very fragile, and the highest compressive strength can reach 5 MPa. After 2 h, the compressive strength of the specimens increased significantly, reaching 16.9 MPa on average. After 8 h of casting, the strength of the specimen continued to increase gradually, reaching 32.7 MPa. At 24 h, it reached 48 MPa. The results showed that the early resistance and microstructure of the specimens were improved continuously during 6–24 h of geopolymerization. The fundamental reason is that the dissolution of alumina and silicon dioxide particles is accelerated in the case of an increased sodium hydroxide concentration. The second stage includes a curing time of 24 h to 7 days. At 48 h, the strength of the specimens increased slightly, with an average of 51.2 MPa, and reached 72.7 MPa at 7 days. The third phase consists of the period from 7 to 28 days. A minimal increase in strength was observed, showing the best ground polymerization after 7 days of curing.

The effect of different plant fibers on the compressive performance of GP composites is different. Gholampour et al. [122] studied the compressive strength of mortar with 1% sisal, coir, hemp, bamboo, and ramie fibers. The GPs with hemp, bamboo, and ramie fibers had a higher compressive strength, while the GPs with coir, sisal, and jute fibers had a lower compressive strength than those of unreinforced GPs. Korniejenko et al. [48] studied the mechanical properties of fly ash-based GPs reinforced with short plant fibers such as coir, cotton, and sisal. One percent of the different types of plant fibers was added to the GP mixture. The results showed that the compressive strength of the GPs containing SF, cotton, and coir fibers was 1.53%, 14.68%, and 26.55% higher than that of pure GP, respectively. In contrast, the compressive strength of the GP reinforced with raffia fibers was 44.87% lower than that of the pure GP. This decrease indicates a lack of bonding between the raffia fibers and the GP paste. Correia et al. [71] studied the effect of adding SFs and pineapple leaf fibers on the mechanical strength of GPs. The results show that a fiber content of 3 vol% hurts the compressive strength of the matrix.

**Figure 8:**
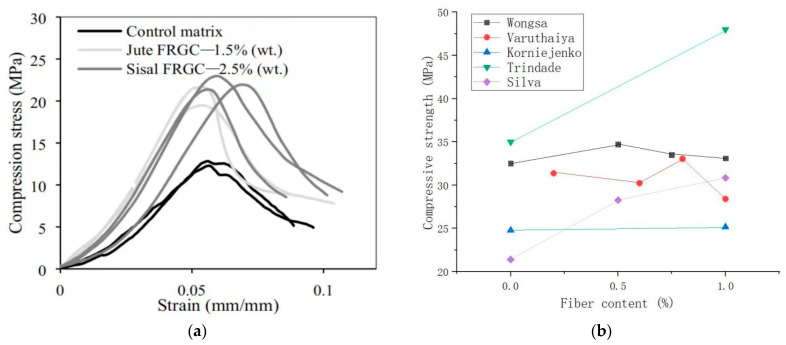
Effect of SFs on compressive strength of SFRGs. (**a**) Compression and strain curves of SF and jute fiber-reinforced GPs. Adapted with permission from [13], copyright 2020 Elsevier; (**b**) relationship between SF content and compressive strength.

The optimum content of different plant fibers in the matrix is also different. Silva et al. [13] found that the addition of sisal and jute fibers resulted in a better performance in the compressive strength for the GPs, as shown in Figure 8a. In the beginning, the compressive strength of the matrix increased with the increase in the fiber content. As can be seen from the figure, the best fiber content for jute fibers was 1.5 wt%, and the best content for SFs was 2.5 wt%. Compared with the control matrix, the elastic modulus of jute and SFRG increased by 103% and 76%, respectively. The presence of these two fibers significantly altered the way the specimen breaks during compression tests. It can also be found in Figure 8a that the optimal content of SFs was greater than that of jute fibers. The results indicated that SFs had better compatibility with the geopolymer matrix than jute fibers. The compressive strength of SFRGs with different SF contents is shown in Figure 8b [13,48,50,51,53]. It can be seen from Figure 8b that SFs had no obvious strengthening effect on the matrix.

#### 4.2.2. Flexural Performance

**Figure 9:**
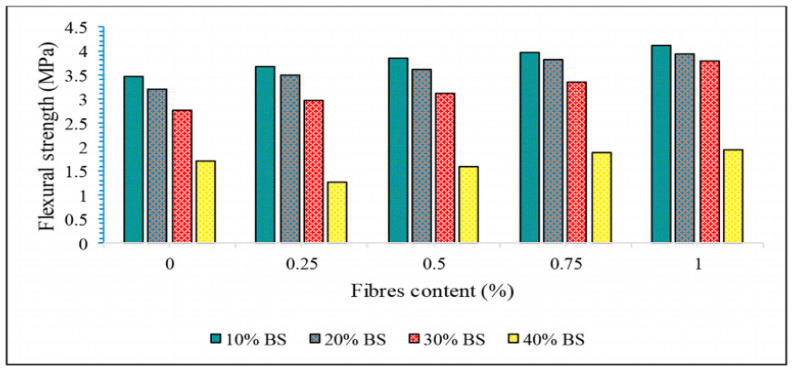
Flexural strength of SFRGs containing SFs and bamboo sticks in different proportions. Adapted with permission from [89], copyright 2021 Elsevier.

Compared with the compressive performance, SFs had a more obvious effect on the flexural strength of GP composites [123,124]. Kavipriya et al. [89] took SFs and bamboo sticks as the main research objects to study their influence on the compressive performance of lightweight GPs. The GP matrix was supplemented with 0.25%, 0.5%, 0.75%, and 1.0% SFs, while the coarse aggregate was replaced with 10%, 20%, and 30% bamboo strips. The results showed that SFs can effectively improve the bending strength of the matrix, as shown in Figure 9. Correia et al. [71] found that the bending strength of SFs and pineapple fibers increased by 111% and 100%, respectively, when a fiber content of 3 vol% was compared with that of ordinary composite materials.

To improve the flexural performance of SFRGs, the optimum content of SFs must be determined. Huang et al. [125] found through experiments that SFs can effectively improve the flexural performance of foamed concrete. It was also found that when the fiber content was less than 0.15%, the higher the SF content, the better the flexural performance and the longer the fatigue life of the foamed concrete. When the content of SFs was greater than 0.15%, the flexural strength and fatigue life of the SFRGs decreased with an increase in SF content. Varuthaiya et al. [53] found that the addition of 0.6% SFs produced the maximum flexural strength of 7.02 MPa on day 28, which was 51% higher than that of the pure GP. According to the test results, when the proportion of SFs exceeded 0.6%, the flexural strength decreased steadily.

The aging effect of the environment will also be reflected in the bending resistance of the composite material. Wei and Meyer [100] measured the 28-day bending property of SF-reinforced mortar. After curing for 28 days under standard conditions, the specimens were subjected to an accelerated dry/wet cycle aging treatment, and the strength values of the specimens were compared for 30 dry/wet cycles. Wei et al. [126] tested the deterioration of embedded SFs using a micro-tensile test and studied the effects of SF strength and composition on the bending properties of the matrix under accelerated aging. Due to fiber degradation, the toughness and flexural strength of the fiber-reinforced mortar after 10 dry/wet cycles decreased by 98% and 90%, respectively. By replacing 20% of the cement with natural diatomaceous earth, the hydration of the cement was enhanced, and 24.4% of the calcium hydroxide was consumed, providing a mild environment for the plant fiber reinforcement. By adding diatomaceous earth, after 10 dry/wet cycles, the toughness and flexural strength reached 7.9 times and 5.3 times that of the pure mortar, respectively.

In general, SFs as a GP reinforcement require a pre-treatment. Different pre-treatment methods have different effects on the bending properties of the composites. Castoldi et al. [81] conducted a three-point bending test with 3 kg/m^3^ of untreated and treated SF-reinforced specimens. Specimens without SFs exhibited a typical brittle material behavior. After the matrix cracked, the stress decreased rapidly, resulting in a brittle fracture of the specimen [127]. The presence of an appropriate amount of fiber changed the bending behavior of the GP matrix [128,129,130]. It was also found that the residual strength of treated and untreated fiber-reinforced cement-based composites after matrix cracking had similar values, indicating that an alkali treatment cannot significantly improve the bending resistance of the SFRG. Trindade et al. [50] observed different loading stages of specimens. The initial phase corresponds to a linear region, indicating that both the fibers and the GP matrix behave elastically. At the initial stage, the properties of the GP determine the stiffness of the matrix, eventually forming the first crack at a later stage. The bending loads of curauá, sisal, and jute fiber-reinforced GPs were 4.96, 5.23, and 6.26 MPa, respectively, which were similar to those of the first crack stresses observed. The stiffness and ultimate strength of the composite were significantly improved after treatment with styrene–butadiene rubber in the GP matrix. This behavior indicates that due to the polymer coating, the fiber matrix bond is increased and its stress transfer mechanism is improved.

**Figure 10:**
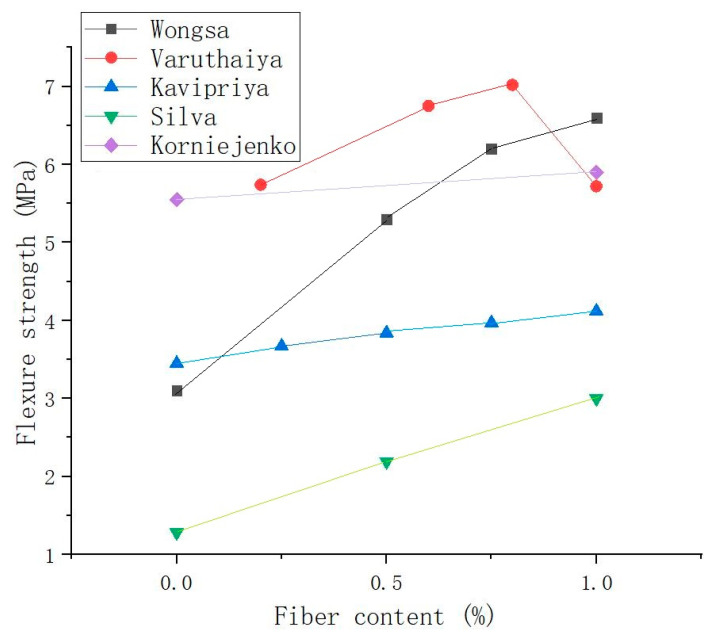
Relationship between fiber content and flexural strength of SFRGs.

Different from the above research results, the flexural strength test results of Korniejenko et al. [48] showed that the properties of the specimens without fibers were the same as those with coconut fibers, cotton fibers, or SFs. Compared with human-made fibers, the effect of plant fibers on the flexural strength of the composite were not particularly significant. Figure 10 shows the relationship between the SF content and flexural performance of SFRGs [13,48,51,53,89]. The bending curve shows that the appropriate amount of fiber was positively related to the bending resistance of the matrix.

#### 4.2.3. Tensile Performance

Fiber strength and interfacial bonds between the fibers and matrix determine the tensile strength of fiber-reinforced GPs. Castoldi et al. [81] carried out pull-out tests on untreated and treated SFs. The SF embedment length was 25 mm. It was found that the pull-out curve of untreated SFs was characterized by fiber desticking. However, for treated SFs, breaking of the SFs was observed after the peak load was reached. SF breakage may be caused by two different mechanisms. In the first mechanism, the debonding load is higher than the tensile strength of the SFs, causing the SFs to collapse. In the second mechanism, the post-bonding, slip-hardening mechanism leads to SF breakage during SF slippage. According to relevant studies, the tensile strength of alkali-treated SFs decreased significantly. However, the interfacial adhesion between SFs and GP matrix was improved, which is related to the removal of some weak components of the SFs after alkali treatment [131,132]. Trindade et al. [50] found that the tensile loads of jute, sisal, and curauá fiber-reinforced GPs were 4.13, 4.37, and 4.83 MPa, respectively. The composite had a higher crack opening, indicating a weaker fiber–matrix bond. This shows that stress transfer between the SFs and GP seems to be more efficient.

Zhou et al. [88] verified that natural fibers such as sisal significantly improved the tensile properties of GPs. Among these test specimens, the tensile strength of the bamboo fiber-reinforced GP was the highest, which was 2.39 MPa. That of the SF-reinforced GP was slightly lower. Due to the high toughness of plant fibers, the fibers in the matrix tend to shrink due to the plastic deformation caused by tensile stress, which can alleviate the extension of the crack tip of the GP matrix. Based on this, the GP specimen did not have obvious relative displacement after tensile cracking failure, but formed filamentous joint cracks through the plant fibers.

**Figure 11:**
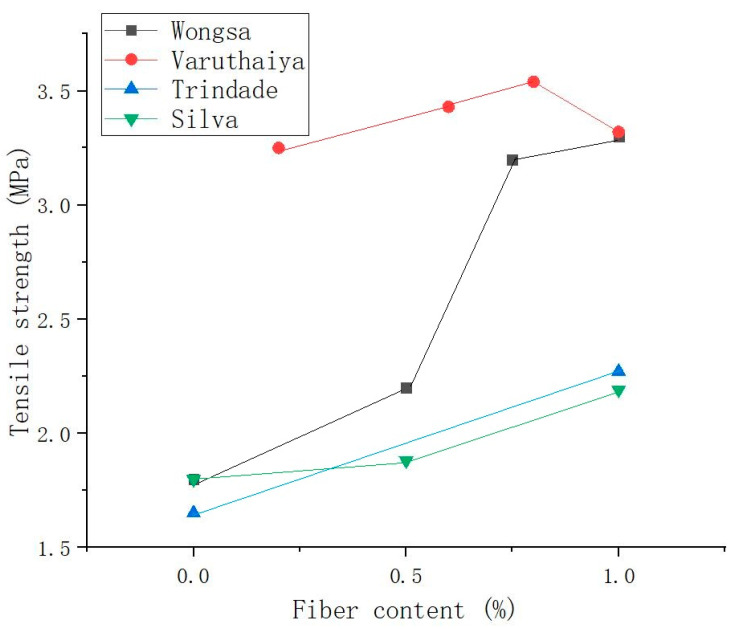
Relationship between fiber content and tensile strength of SFRGs.

Varuthaiya et al. [53] found that adding 0.6% SFs to the matrix produced a maximum fission tensile strength of 3.54 MPa, which was 20% higher than that of the matrix without SFs. According to the test results, increasing the proportion of isotropic fibers to 0.6% can increase the splitting tensile strength, but otherwise, the splitting tensile strength steadily decreased. Gholampour et al. [122] verified that under the given binder and sand type, 1% content of sisal and ramie fibers both increased the matrix tensile strength. When the ramie fiber content increased to 2%, the direct tensile strength of the GP decreased. Among all the fiber-reinforced GPs, the 1% ramie fiber-reinforced GP had the highest tensile strength. This good performance is due to the bridging between the fibers in the cracks of the GP matrix. However, the agglomeration of plant fibers in the matrix leads to a lack of interfacial adhesion between the fibers and the matrix, which leads to a decrease in GP strength. Figure 11 shows the curve of the relationship between SF content and the tensile strength of SFRGs [50,51,53]. The results show that the tensile strength of the SFRG is positively correlated with the content of SFs when the content of the fibers is low in the matrix.

Of course, SF is a representative plant fiber, and its durability and compatibility with GP matrix are a focus of researchers [133,134]. One of the ways to improve the durability of plant fibers is to increase fiber–matrix adhesion by changing the composition of the matrix or changing the alkalinity of the matrix [135,136,137]. By modifying the plant fibers, we can improve their ability to resist alkaline attacks and improve their compatibility with matrix. As the tension in the interfacial region between the fibers and matrix is transferred from the matrix to the fibers through interfacial bonding, the effective interfacial interaction between the fibers and the matrix is significantly increased. To alleviate fiber degradation and improve the weak bond between the fibers and matrix interface, the fibers can be modified by physical and chemical methods. These treatments alter the fiber surface by binding active functional groups to the active groups of the matrix. As a result, the hydrophobic fibers produced by these treatments have a greater fiber surface roughness and stronger matrix affinity [138,139,140]. In some special types of lignocellulosic fibers, the lignin can be completely degraded by alkali in the GP matrix, resulting in a serious reduction in the GP’s tensile performance [141]. In addition, the keratosis of fibers affects the mechanical behavior of the fibers. Keratinization promotes more fiber–matrix bonds and also improves the friction mechanism. The crystallization of cellulose and the formation of polymer chains in microfibrils lead to the improvement of the GP’s tensile properties, but the number of cycles may not cause structural damage to such bonds. Ferreira et al. [142] investigated the effects of keratosis on the mechanical behavior of sisal, jute, and curauá fibers. This conclusion was confirmed by wetting and drying the fibers about 5 and 10 times.

In fact, from a sustainability perspective, concrete manufacturing accounts for 5% of global carbon emissions, which is a huge amount. By comparing the carbon emissions of SFRGs with that of traditional concrete, GP materials have obvious environmental benefits. A compact and uniform interfacial structure is formed between plant fibers and GP. This interface structure can not only effectively transfer stress and prevent crack expansion, but also improve the resistance of the material to external environmental factors. At the same time, more contact points and frictional hindrance effects are formed at the interface, which further increase the overall stiffness and strength of the composite. Although there are many research cases of fiber-reinforced geopolymers, there are few engineering applications. With the deepening of research, based on the concept of carbon peaking and carbon neutrality, their engineering application will be further expanded. It is necessary to consider the effects of different types and length ratios on the final properties of composite materials when optimizing the design at the microscopic level. For example, selecting the appropriate length ratio to achieve the best load transfer effect; adjusting the surface treatment method to promote the interface adhesion; and controlling the amount of additives to avoid too much or too little additive, resulting in unbalanced effects. In short, the use of plant fibers to strengthen GPs has many advantages at the microscopic scale and the design can be optimized for different needs, so composite materials can have a wider and more important application prospect in the field of engineering.

## 5. Conclusions

In this paper, the relevant studies on SFRG composites in recent years were reviewed, which provides some valuable information on the progress of this kind of research. In terms of mechanical properties, due to the characteristics of SFs, the addition of SFs into the matrix can make up for the characteristics of easy cracking and high brittleness of GPs.

Compared with other fibers, the cellulose content of SFs is second only to cotton fibers, and is higher than that of wheat straw fibers, bamboo fibers, and coir fibers.

At the microscopic level, more contact points and frictional hindrance effects are formed at the interface between SFs and GPs, which further increases the overall stiffness and strength of the composite. The content of plant fiber needed in the GP matrix is not too high, the general content is 0.5–3%; through the compression test, the best content for jute fibers is 1.5 wt%, and the best content for SFs is larger, up to 2.5 wt%. This shows that the compatibility between SFs and the GP matrix is better than that of jute fibers.

With the increase in fiber reinforcement content, the strength of the matrix has a certain limit. The increase in compressive strength of the matrix due to plant fibers is not obvious. In the compressive strength test, the strengthening effect of coir fibers was the most obvious, which increased by 1.5%. SFs can effectively improve the flexural and tensile properties of GPs. However, compared with bamboo fibers, the tensile strength of SFRGs is 46% lower than that of bamboo fiber-reinforced GPs.

## Figures and Tables

**Table 1:** The physical properties of SFs for GP reinforcement.

Length/mm	Diameter/μm	Density/g·cm^−3^	Modulus/GPa	Tensile Strength/MPa	Elongation/%	Ref.
3	500	-	9.0–38.0	363–700	2.0–7.0	[48]
-	230–250	0.90	19.0	577	-	[49]
20	-	1.30	15.1	705	-	[50]
35–40	179	1.45	-	-	-	[51]
-	-	1.51	11.5	372	0.6	[52]
60	750	1.45	9.4–15.8	568–640	2.0–3.0	[53]
30	-	1.13	12.4	371	-	[54]
-	-	1.45	9.0–20.0	400–700	5.0–14.0	[55]
180–600	-	-	-	31–221	14.8	[56]
12	-	1.40	-	560	-	[57]
6	140–200	1.45	17.0–22.0	530–630	-	[58]

**Table 2:** The properties of other plant fibers used for GP reinforcement.

Plant Fiber	Length/mm	Diameter/μm	Density/g·cm^−3^	Modulus/GPa	Tensile Strength/MPa	Elongation/%	Ref.
Cotton	30	1000	-	4.8	400	-	[48]
Palm	3	500	-	30.0	500	2.0–4.0	
Coir	3	500	-	2.2–6.0	95–230	15.0–51.4	
Jute	20	-	1.5	6.16	104	-	[50]
Curauá	20	-	1.4	27.8	1205	-	
Coconut	35–40	1170	1.2	-	-	-	[51]
Bamboo	-	300–380	1.15	5.96	518	10.04	[59]
Hemp	0.5–8	-	1.4–1.5	23.5–90	270–900	1.0–3.5	[60]

**Table 3:** Designs of mix ratio of SFRGs [48,49,50,51,52,53,88,89].

Authors	Precursor		Alkaline Activator		Fiber Content/%
	Composition	Specification Feature	Composition	Specification Feature	
Korniejenko et al.	Fly ash	60% of the particles are smaller than 56 μm	NaOH; sodium silicate solution	NaOH 8M; water glass 2.5	1.0
Santos et al.	Sludge; Portland Cement	Sludge 34%; cement 7%	KOH and silicon dioxide	KOH 9%; water 15% SiO_2_ 12%	2.0
Kavipriya et al.	Fly ash	Superplasticizers 1%	NaOH and sodium silicate	Water glass 2.0; NaOH 10 M; fly ash/activator 0.67	0.25; 0.5; 0.75; 1.0
Trindade et al.	Metakaolin	The average particle size is 15 μm	NaOH and sodium silicate	The binder/aggregate weight ratio is 1:1	3.0
Wongsa et al.	High-lime fly ash	The fineness of fly ash is 59%	NaOH and sodium silicate	NaOH 10 M, sodium silicate (sodium oxide = 12.53%, silica 30.24%, water 57.23%)	0.5; 0.75; 1.0 *
Zhou et al.	Fly ash; Slag (GGBS)	Fly ash and GGBS powder composition ratio is 1:1	NaOH and sodium silicate	Water 65.3%, Na_2_SiO_3_ 24.8%, and NaOH 9.9%	1.0
Alves et al.	Metakaolin	The average particle size is 12 μm	NaOH and sodium silicate	Activator/kaolin 0.352, 0.41, 0.55, 0.69, 0.748	0.85; 3.0; 5.15; 6.0
Varuthaiya et al.	Low-lime F class fly ash	Finesses modulus 7.86	NaOH and sodium silicate	The mass ratio of Na_2_SiO_3_/NaOH: 2.5	0.2, 0.6, 0.8. 1.0

The * indicates the mass content, the other is the volume content.

**Table 4:** Main technical indexes for preparation of SFRGs.

Mixing Method	Specimen Size/mm	Initial Setting Conditions	Curing Conditions	Test Content	Ref.
Mechanical mixer	50 × 50 × 50; 200 × 50 × 50	Heat in a laboratory dryer at 75 °C for 24 h	28 days at room temperature	Compression; bending	[48]
Mechanical mixer	50 × 195	Soak in 22 ± 2 °C water for 1 day, then dry in an oven at 40 ± 2 °C for 2 days	About 6 months	Compression; bending	[49]
Planetary mixer	Cylinder: 100 × 50; 450 × 60 × 12	At room temperature (22 ± 3 °C)	At 2, 4, 8, 24, and 48 h, 7, 14, and 28 days	Bending; tensile force	[50]
Mechanical mixer	Cylinder: 200 × 100; 40 × 40 × 160	In an oven at 60 °C for 48 h	At 50% RH and 25 °C	Compression; bending; tensile force	[51]
Mechanical mixer	170 length by 40 wide	Room temperature	At room temperature (25 ± 5 °C)	Compression; bending	[52]
Mechanical mixer	150 × 150 × 150; 100 × 100× 500; cylinder: 150 × 300/150 × 100	60 °C steam for 24 h	28 days	Compression; bending; tensile force	[53]
Mechanical mixer	40 × 40 × 160; dog-bone mold	23 ± 5 °C Standard curing tank	Standard curing tank for 7, 14, and 28 days	Compression; tensile force	[88]
Mechanical mixer	500 × 100 × 100	Environmental model	Environmental curing for 7, 14, and 28 days	Bending	[89]

## Data Availability

Data will be made available on request.
